# Detection, Measurement, and Enhancement of Happiness

**DOI:** 10.1155/2014/841206

**Published:** 2014-05-20

**Authors:** Jhing-Fa Wang, Chung-Hsien Wu, Shulan Hsieh, Shyhnan Liou, Bo-Wei Chen

**Affiliations:** ^1^Department of Electrical Engineering, National Cheng Kung University, No. 1 Daxue Road, Tainan 70101, Taiwan; ^2^Department of Computer Science and Information Engineering, National Cheng Kung University, No. 1 Daxue Road, Tainan 70101, Taiwan; ^3^Department of Psychology and Institute of Allied Health Sciences, National Cheng Kung University, No. 1 Daxue Road, Tainan 70101, Taiwan; ^4^Institute of Creative Industries Design, National Cheng Kung University, No. 1 Daxue Road, Tainan 70101, Taiwan

Research on happiness has gained much attention in recent years since the United Nations published a report on the worldwide happiness index. Such publication subsequently stimulates academia and national policies; related research therefore becomes a hot spot. The idea of happiness research is dated back to 1972 when the king of Bhutan proposed using Gross National Happiness to replace traditional economic indices. The pursuit of well-being happiness gradually initiates the formation of new theories, for example, Satisfaction with Life Index, Quality-of-life Index, Happy Planet Index, and positive psychology.

Although a great number of studies have been conducted in social science, there are still few papers accommodating the needs of technology. In view of this, the special issue particularly highlights happiness research on information technology. More specifically, this issue discusses how technology can be used for enhancement of positive emotions, not simply for detection and measurement.

Five papers selected from the submission present a recent update and the advances of technologies in detection, measurement, and enhancement of happiness. The paper by Y. Chin et al. focused on kernel design for Support Vector Machines (SVMs). The authors used music classification as a case study to evaluate the proposed SVM. Their research helped users select music of interest based on emotions in the audio. C. Chuang et al. developed a digital learning system based on somatosensors. Such a system was capable of enhancing the learning performance of users by analyzing physical expressions and interactions. The work by C. Lin et al. investigated feature extraction for recognition of emotional speech. The recognition result could be further evaluated by medical doctors or psychologists and subsequently made into personal profiles. N. Jatupaiboon et al. proposed an emotion classification system based on real-time electroencephalograms (EEGs). The brain waves of subjects were elicited by pictures and sound and subsequently analyzed by the proposed spectral characterization. The authors also constructed a feedback system that allowed users to practice controlling emotions. Finally, P. Cipresso et al. created a multidimensional valence-arousal model to present emotional changing paths. Their model demonstrated another research direction in psychophysiology and affective computing.

Through these papers, readers can obtain an overview of happiness research on information technology. Besides, readers can also understand several practical issues, including feature extraction, pattern recognition, and feedback analysis during processing audiovisual and biomedical signals. In the near future, we expect the happiness research on information technology will become a new subject, “Happiness Informatics,” and have an impact on society.

*Jhing-Fa Wang* *Jhing-Fa Wang* *Chung-Hsien Wu* *Chung-Hsien Wu* *Shulan Hsieh* *Shulan Hsieh* *Shyhnan Liou* *Shyhnan Liou* *Bo-Wei Chen* *Bo-Wei Chen*

